# The Vacuolar Zinc Transporter TgZnT Protects Toxoplasma gondii from Zinc Toxicity

**DOI:** 10.1128/mSphere.00086-19

**Published:** 2019-05-22

**Authors:** Nathan M. Chasen, Andrew J. Stasic, Beejan Asady, Isabelle Coppens, Silvia N. J. Moreno

**Affiliations:** a Center for Tropical and Emerging Global Diseases, University of Georgia, Athens, Georgia, USA; b Department of Infectious Diseases, University of Georgia, Athens, Georgia, USA; c Department of Microbiology, University of Georgia, Athens, Georgia, USA; d Department of Molecular Microbiology and Immunology, Johns Hopkins University Bloomberg School of Public Health, Baltimore, Maryland, USA; e Department of Cellular Biology, University of Georgia, Athens, Georgia, USA; University at Buffalo

**Keywords:** *Toxoplasma gondii*, zinc transport, transporters

## Abstract

Zinc (Zn^2+^) is the most abundant biological metal ion aside from iron and is an essential element in numerous biological systems, acting as a cofactor for a large number of enzymes and regulatory proteins. Zn^2+^ must be tightly regulated, as both the deficiency and overabundance of intracellular free Zn^2+^ are harmful to cells. Zn^2+^ transporters (ZnTs) play important functions in cells by reducing intracellular Zn^2+^ levels by transporting the ion out of the cytoplasm. We characterized a Toxoplasma gondii gene (TgGT1_251630, TgZnT), which is annotated as the only ZnT family Zn^2+^ transporter in T. gondii. TgZnT localizes to novel vesicles that fuse with the plant-like vacuole (PLV), an endosome-like organelle. Mutant parasites lacking TgZnT exhibit reduced viability in *in vitro* assays. This phenotype was exacerbated by increasing zinc concentrations in the extracellular media and was rescued by media with reduced zinc. Heterologous expression of TgZnT in a Zn^2+^-sensitive Saccharomyces cerevisiae yeast strain partially restored growth in media with higher Zn^2+^ concentrations. These results suggest that TgZnT transports Zn^2+^ into the PLV and plays an important role in the Zn^2+^ tolerance of T. gondii extracellular tachyzoites.

**IMPORTANCE** Toxoplasma gondii is an intracellular pathogen of human and animals. T. gondii pathogenesis is associated with its lytic cycle, which involves invasion, replication, egress out of the host cell, and invasion of a new one. T. gondii must be able to tolerate abrupt changes in the composition of the surrounding milieu as it progresses through its lytic cycle. We report the characterization of a Zn^2+^ transporter of T. gondii (TgZnT) that is important for parasite growth. TgZnT restored Zn^2+^ tolerance in yeast mutants that were unable to grow in media with high concentrations of Zn^2+^. We propose that TgZnT plays a role in Zn^2+^ homeostasis during the T. gondii lytic cycle.

## Abstract (precis)

Toxoplasma gondii is an intracellular pathogen of human and animals. T. gondii pathogenesis is associated with its lytic cycle, which involves invasion, replication, egress out of the host cell, and invasion of a new one. T. gondii must be able to tolerate abrupt changes in the composition of the surrounding milieu as it progresses through its lytic cycle. We report the characterization of a Zn^2+^ transporter of T. gondii (TgZnT) that is important for parasite growth. TgZnT restored Zn^2+^ tolerance in yeast mutants that were unable to grow in media with high concentrations of Zn^2+^. We propose that TgZnT plays a role in Zn^2+^ homeostasis during the T. gondii lytic cycle.

## INTRODUCTION

Toxoplasma gondii is an apicomplexan parasite and is an important cause of congenital disease and infection in immunocompromised patients. The parasite can cause ocular uveitis in immunocompetent individuals (1), pneumonia or encephalitis in immune-deficient patients (2), and serious malformations in congenitally infected children (3). The pathogenesis of T. gondii is linked to its lytic cycle, which comprises secretion of adhesins from specific secretory organelles, invasion, intracellular replication, and egress. T. gondii replicates exclusively inside a host cell, where it resides inside a parasitophorous vacuole (PV) (4). The PV membrane allows the host cytosolic ions to equilibrate with the lumen of the vacuole, and upon exit, *Toxoplasma* is exposed to dramatic changes in its surrounding ionic and nutrient milieu. We previously characterized a lysosomal compartment, termed the plant-like vacuole (PLV) or VAC, and proposed an important role for this organelle in controlling ionic stress during the short extracellular phase of the parasite (5, 6). The PLV becomes prominent when *Toxoplasma* is extracellular and its proton pumps (7, 8) create a proton gradient that is used for the countertransport of Ca^2+^ (5) and other ions.

The zinc ion (Zn^2+^) must be tightly regulated because both a deficiency and an excess of cytoplasmic free Zn^2+^ are deleterious for cells (9–11). Zinc is an essential element that acts as a cofactor for a large number of enzymes and regulatory proteins and that also participates in cell signaling (12, 13). More than 300 enzymes that utilize Zn^2+^ have been identified across all enzyme classes and phyla (14). Notably, 3 to 10% of the genes encoded by the human genome, over 3,000 in total, are thought to encode proteins that interact with Zn^2+^, a number that is likely underestimated because new Zn^2+^-protein interactions are still being discovered (15–17).

Enzyme inhibition, disruption of protein folding, and induction of apoptosis are some of the proposed mechanisms by which high concentrations of Zn^2+^ may be deleterious to cells (9–11). The consistent abundance of Zn^2+^ in our environment during the evolution of life has introduced a selective pressure on all living organisms to evolve complex mechanisms to regulate total cellular Zn^2+^ and intracellular free Zn^2+^. The total concentration of cellular zinc in eukaryotic cells typically ranges from 0.1 to 0.5 mM (18); however, most of the Zn^2+^ in cells is bound to proteins and sequestered into so-called zincosomes (19) or lysosomal compartments. The resting intracellular free Zn^2+^ concentration is reported to be at picomolar levels (20), and cytosolic zinc-binding proteins exhibit an affinity for Zn^2+^ in the picomolar range (21, 22). These picomolar concentrations represent less than 0.0001% of total cellular Zn^2+^, exemplifying the precise control of cytoplasmic free Zn^2+^ in eukaryotic cells. Free Zn^2+^ in the extracellular space was reported to be in the range of 5 to 25 nM in the central nervous system (23), which is more than 1,000-fold higher than the predicted intracellular concentration.

T. gondii is exposed to sharp changes of extracellular Zn^2+^ upon egress, and we propose that the PLV plays an important role in the ability of *Toxoplasma* to efficiently survive these changes. In the present work, we characterized a ZnT family Zn^2+^ transporter (TgGT1_251630, TgZnT). We localized TgZnT, characterized the phenotypic profile of conditional knockdown mutants, and used *TgZnT* to rescue Zn^2+^ tolerance in a Zn^2+^-intolerant Saccharomyces cerevisiae yeast mutant.

## RESULTS

### Identification of a Zn^2+^ transporter in T. gondii.

**FIG 1:**
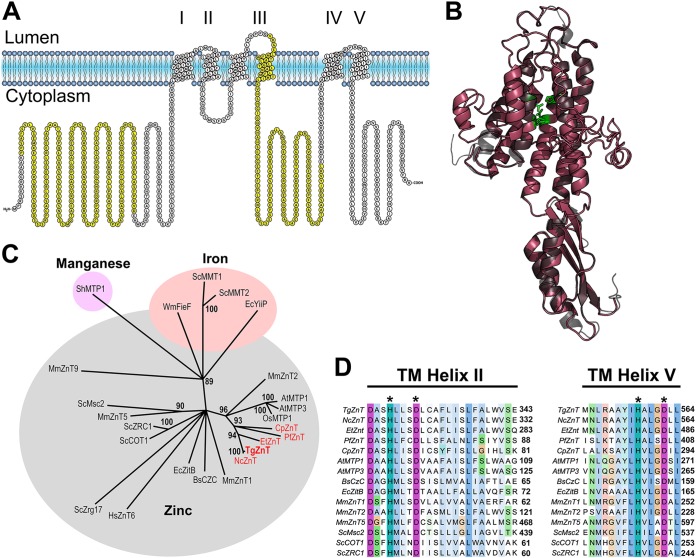
Sequence analysis of TgZnT. (A) Protter topology analysis of the TgZnT predicts for 6 transmembrane domains, which is typical of ZnT family transporters. Areas highlighted in yellow are regions used for polyclonal antibody production (Fig. 3). (B) Phyre2 modeling of TgZnT (red) shows a predicted structure similar to that of the E. coli ZnT transporter YiiP (gray). Side chains of the predicted Zn^2+^ binding residues in transmembrane helices II and V are shown in green. (C) Unrooted tree of TgZnT, apicomplexan orthologs, and other ZnTs. The branch to which TgZnT (red, bold) and its apicomplexan orthologs (red) belongs is the ZnT2-like subfamily, which primarily transports Zn^2+^. (D) Multiple-sequence alignment of transmembrane (TM) helices II and V of TgZnT and its apicomplexan orthologs with various other ZnTs that transport Zn^2+^. The histidines and aspartic acid residues that are predicted to be part of the intramembrane Zn^2+^-binding site (*) are conserved in TgZnT and its apicomplexan orthologs. Cp, Cryptosporidium parvum; Pf, Plasmodium falciparum; Et, Eimeria tenella; Nc, Neospora caninum; Os, Oryza sativa; At, Arabidopsis thaliana; Hs, Homo sapiens; Sh, Stylosanthes hamata; Mm, Mus musculus; Sc, Saccharomyces cerevisiae; Bs, Bacillus subtilis; Wm, Wautersia metallidurans; Ec, Escherichia coli.

With the aim of characterizing the potential role of the PLV in the survival and thriving of *Toxoplasma* during its extracellular passage, an essential phase of its lytic cycle, we looked at potential transporters that localize to the PLV and that could function in the transport of ions for which a strict control is required. One of these ions, Zn^2+^, was especially interesting because of several reasons. First, Zn^2+^ levels need to be tightly controlled; second, there was proteomic evidence for the presence of a zinc transporter in *Toxoplasma* and in a PLV-enriched fraction (ToxoDB and unpublished data); and third, evidence for the proton gradient needed for its function was demonstrated in previous work (5). The Zn^2+^ transporter gene annotated in ToxoDB (TgGT1_251630) predicts the expression of a protein of 715 amino acids with a predicted molecular weight of 77 kDa and an isoelectric point of 5.86. We named the gene *TgZnT* because it is the single member of this family of Zn^2+^ transporters annotated in the T. gondii genome. The *TgZnT* gene product is predicted to contain 6 transmembrane domains (Fig. 1A), forming a structure similar to that of the Escherichia coli Zn^2+^ transporter YiiP (Fig. 1B).

We studied the phylogenetic profile of *TgZnT*, and for this we generated a bootstrapped neighbor-joining tree of aligned and trimmed sequences (see Fig. S1 in the supplemental material) of various ZnT family proteins from a variety of organisms as well as TgZnT and its apicomplexan orthologs (Fig. 1C). The tree analysis showed that TgZnT groups with the ZnT-2 family of plant and mammalian Zn^2+^ transporters (24) along with orthologs in other apicomplexan parasites (including both coccidian and hemosporidian parasites) (Fig. 1C). This grouping suggests that TgZnT and its orthologs may have derived from a single gene in a distant common ancestor of plants, mammals, and apicomplexans. TgZnT also possesses the histidine and aspartic acid residues thought to be required for intramembrane Zn^2+^ binding in transmembrane helixes II and V (Fig. 1D).

10.1128/mSphere.00086-19.1 FIG S1 Trimmed alignment used for phylogenetic analysis. The sequences utilized are shown in Table S1 in the supplemental material. Trimming was performed manually to remove large gap regions after sequence alignment. Download FIG S1, TIF file, 2.9 MB. Copyright © 2019 Chasen et al. 2019 Chasen et al. This content is distributed under the terms of the Creative Commons Attribution 4.0 International license.

10.1128/mSphere.00086-19.3 TABLE S1 Amino acid sequences used for alignments and tree generation. Download Table S1, PDF file, 0.06 MB. Copyright © 2019 Chasen et al. 2019 Chasen et al. This content is distributed under the terms of the Creative Commons Attribution 4.0 International license.

### TgZnT-HA localizes to the plant-like vacuole and to cytoplasmic vesicles.

**FIG 2:**
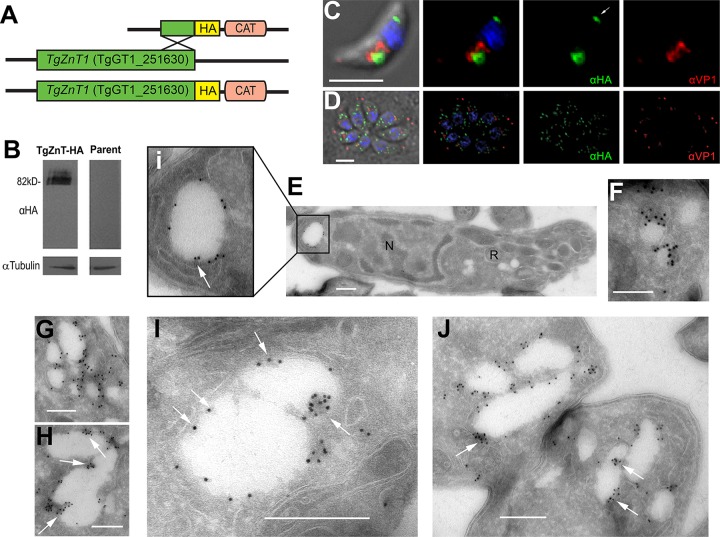
C-terminal tagging of TgZnT and its localization to intracellular vesicles and the PLV. (A) Scheme showing the modified *TgZnT* locus (green), C-terminal HA tag (yellow), and selection marker (chloramphenicol acetyltransferase [CAT]; pink). (B) Western blot analysis of lysates obtained from tachyzoites of the *ku80* (parent) and *TgZnT-HA* clonal cell lines showing bands of the predicted molecular weight (82 kDa). Tubulin was used as a loading control. (C) Immunofluorescence assay (IFA) of an extracellular *TgZnT*-*HA* tachyzoite showing the partial colocalization of anti-HA (1:50; green) and anti-VP1 (1:2,000; red), a PLV marker. Labeling in the posterior compartment (arrow) was also observed. (D) IFA of intracellular *TgZnT*-*HA* tachyzoites showing labeling with anti-HA (1:50; TgZnT; green) and anti-VP1 (1:2,000; red). (E to I) Cryo-immuno electron microscopy (CryoIEM) of an extracellular tachyzoite using anti-HA antibodies shows that TgZnT-HA localizes to a posterior vacuole (Ei), small vesicles (F), large vesicles (G, I), and PLV structures (H, J) in extracellular tachyzoites. TgZnT-HA often localizes to invaginations of the PLV-like structures and larger vacuoles (arrows, Ei, H, and I), suggesting the potential fusion of TgZnT vesicles. CryoIEM was labeled with immunogold (10-nm beads). N, nucleus; R, rhoptry. Bars, 3 μm (C and D), 500 nm (E and G to J), and 100 nm (F).

To investigate the localization of TgZnT, the TgGT1_251630 gene was endogenously tagged with a 3× hemagglutinin (3×HA) tag at its 3′ end, using the ligation-independent cloning C-terminal tagging plasmid previously described (25). This approach avoids the overexpression and potential abnormal distribution of the tagged protein. Western blot analysis of a clonal parasite line expressing TgZnT-HA showed several bands around the predicted molecular weight of TgZnT plus the additional 4 kDa of the 3×HA tag (∼82 kDa) (Fig. 2B). The presence of multiple bands suggests that TgZnT is posttranslationally modified, which is additionally supported by the prediction of phosphorylation and methylation sites annotated in the EuPathDB (26) entry for TgZnT (TgGT1_251630) (27, 28). Immunofluorescence analysis with a clonal parasite line expressing TgZnT-HA showed different distributions of the labeling in extracellular and intracellular tachyzoites (Fig. 2C and D). In extracellular tachyzoites, TgZnT-HA localized to two prominent vacuoles, one apical and one posterior. The apical vacuole showed partial colocalization with the vacuolar-H^+^-pyrophosphatase, a PLV marker (anti-VP1) (Fig. 2C). In intracellular tachyzoites, TgZnT-HA localized to dispersed vesicles throughout the cytoplasm which did not colocalize with the anti-VP1 labeling (Fig. 2D). We performed cryo-immuno electron microscopy (CryoIEM) of *TgZnT-HA* extracellular tachyzoites to obtain fine details of the TgZnT localization (Fig. 2E to J). Gold particle labeling was observed in structures ranging from small vesicles (∼100 nm) (Fig. 2F) to large vacuoles (>250 nm) (Fig. 2E, G to J). Of particular note, we saw that labeling favored the invaginations into the larger vacuoles (Fig. 2Ei, H to J, arrows).

**FIG 3:**
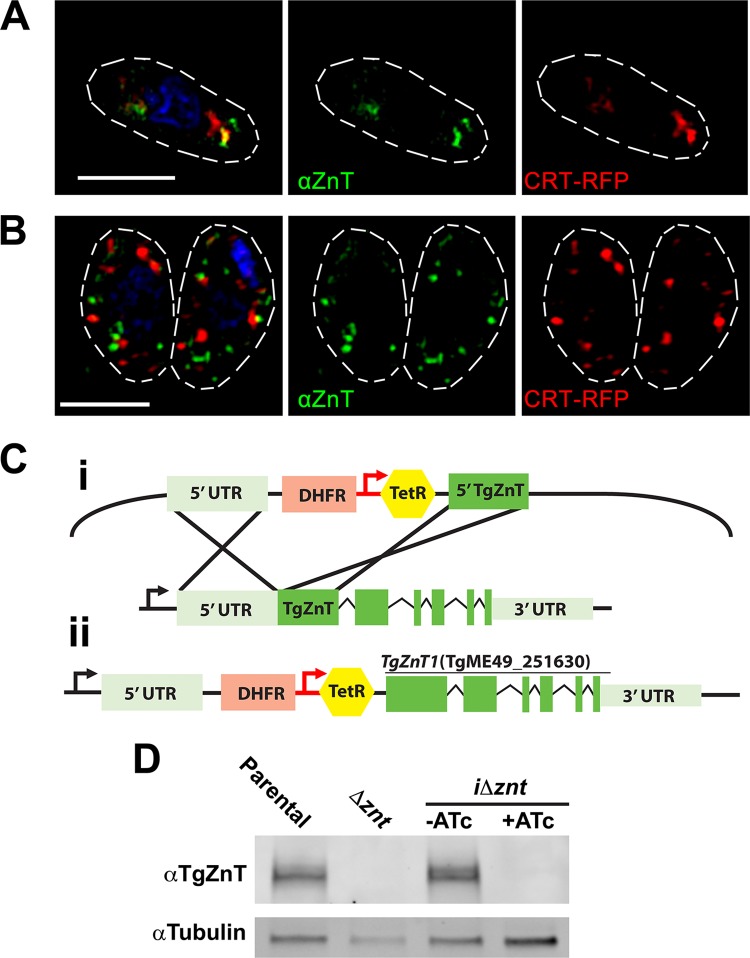
Localization of TgZnT with specific mouse antibodies and generation of conditional knockdown mutants. (A) IFA of an RH extracellular tachyzoite showing the partial colocalization of polyclonal mouse anti-TgZnT (αZnT) with the PLV marker CRT-RFP. (B) IFA of intracellular tachyzoites showing anti-TgZnT labeling of vesicles throughout the tachyzoite, excluding the nucleus. There was no colocalization with the PLV marker CRT-RFP. (C) (i) Strategy for insertion of the tet7sag4 promoter (red arrow) into the *TgZnT* endogenous locus using CRISPR/Cas9. (ii) Final inducible knockdown locus (*i*Δ*znt*) showing the endogenous promoter (black arrow) and the 5′ UTR (light green) displaced by the DHFR selection cassette (pink) and the tet7sag4 promoter (red) with the tetracycline (Tet) repressor (yellow), followed by the coding region of *TgZnT* with exons (green). (D) Western blot analysis of lysates from the parental strain (the Δ*ku80 TATi* strain) and the Δ*znt* and *i*Δ*znt* mutants after growth with or without 0.5 μg anhydrotetracycline (ATc). Lysate from *i*Δ*znt* tachyzoites after growth in ATc did not show labeling with anti-TgZnT. Tubulin was used as a loading control.

To investigate the localization of untagged, wild-type TgZnT, we generated specific antibodies against a fusion of two TgZnT loop domains (Fig. 1A, yellow) in mice. Western blot analysis of lysates from RH tachyzoites showed several bands around the expected molecular weight of 77 kDa (Fig. 3D), similar to what was observed with TgZnT-HA (Fig. 2B). We also performed immunofluorescence assays (IFAs) using polyclonal anti-TgZnT, which showed the labeling of two large vacuoles in extracellular tachyzoites, and one of them showed colocalization with the red fluorescent protein (RFP)-tagged chloroquine resistance transporter (CRT), a PLV marker (Fig. 3A) (29). In intracellular tachyzoites we observed a dispersed vesicular localization (Fig. 3B) that was also seen in the IFAs of TgZnT-HA tachyzoites. These vesicles did not colocalize with the vesicles labeled by CRT-RFP.

### TgZnT knockout mutants exhibit reduced growth in the presence of extracellular Zn^2+^.

To establish the role of TgZnT in the T. gondii lytic cycle, we first generated knockout mutants by inserting a dihydrofolate reductase (DHFR) resistance selection cassette at the beginning of exon 1 using the CRISPR/Cas9 system and a protospacer for this region of the gene (Fig. S2A). A Western blot analysis of lysates from a subclone (the Δ*znt* clone) of the resulting mutants showed the absence of anti-TgZnT labeling, suggesting gene disruption (Fig. S2B). We complemented these mutants with a copy of *TgZnT* in an overexpression vector utilizing the tubulin promoter (pDTM3) (30). These clones (the Δ*znt-ZnT* clones) overexpressed TgZnT, as was seen by Western blot analysis of their lysates (Fig. S2B). Immunofluorescence assays of parental strain RH and the knockout and complemented overexpressing mutants confirmed these results (Fig. S2C). The knockout mutants (the Δ*znt* mutants) showed reduced growth in plaque assays (Fig. S2D), but the overexpression of TgZnT in the Δ*znt-ZnT* clones was also deleterious for growth, and it was not possible to complement the growth phenotype of the Δ*znt* mutant.

10.1128/mSphere.00086-19.2 FIG S2 Both gene disruption and overexpression of TgZnT affect *Toxoplasma* growth *in vitro*. (A) Scheme showing the insertion of a DHFR cassette (pink) into *TgZnT* exon 1 using the CRISPR/Cas9 system. The endogenous promoter (arrow), untranslated 5′ UTR and 3′ UTR (light green), and exons (green) are indicated. (B) Western blot analysis of lysates from the RH (parental), Δ*znt*, and Δ*znt-ZnT* (complemented with an exogenous copy of TgZnT under the control of the tubulin promoter) strains with anti-TgZnT showing a reduction in the labeling in the Δ*znt* mutant lysate and a remarkable increase of labeling in the Δ*znt-ZnT* mutant lysate. Tubulin was used as a loading control. (C) IFA of intracellular tachyzoites of parental, Δ*znt*, and Δ*znt-ZnT* tachyzoites, showing the absence of labeling in Δ*znt* mutants and excessive labeling in the Δ*znt-ZnT* mutants, which overexpress TgZnT. The minor labeling that remains in the Δ*znt* tachyzoites is most likely nonspecific labeling by the polyclonal anti-TgZnT. Exposure and display conditions were identical for the IFA images. (D) Plaque assay of the RH parental strain and the Δ*znt* and Δ*znt-ZnT* mutants showing reduced growth in the Δ*znt* and Δ*znt-ZnT* mutants. The reduced-growth phenotype observed in the Δ*znt-ZnT* mutant is even greater than that observed in the Δ*znt* mutants, suggesting that overexpression of TgZnT is detrimental to progression of the tachyzoite lytic cycle. Download FIG S2, PDF file, 0.8 MB. Copyright © 2019 Chasen et al. 2019 Chasen et al. This content is distributed under the terms of the Creative Commons Attribution 4.0 International license.

The effect of overexpression of *TgZnT* on parasite growth did not permit proper analysis of the specific biological functions of *TgZnT*, so we next created conditional mutants for *TgZnT*, which allowed for controlled expression of the gene. For this, we modified the endogenous *TgZnT* locus by inserting a tet7sag4 promoter at the 5′ end of the predicted open reading frame (ORF). This element responds to anhydrotetracycline (ATc) by repressing expression of the downstream gene (Fig. 3C). Subclones (the final inducible knockdown locus [*i*Δ*znt*] clones) were isolated, and Western blot analysis of lysates from these clonal lines revealed that expression was responsive to ATc (Fig. 3D).

**FIG 4:**
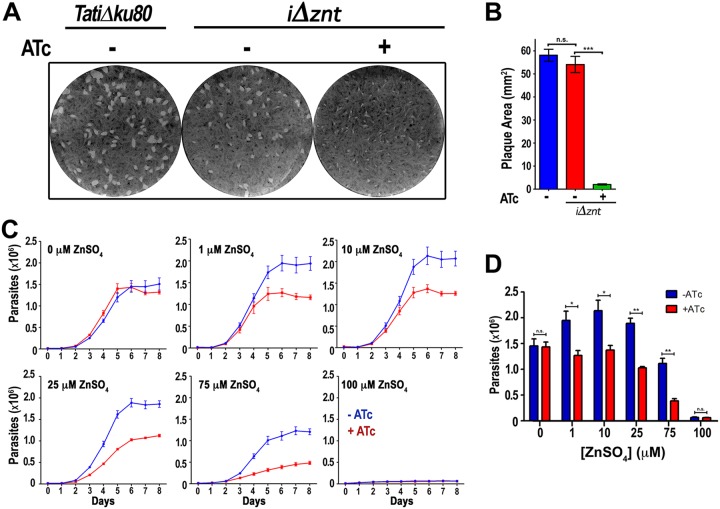
Knockdown of TgZnT results in reduced *in vitro* growth of *Toxoplasma*. (A) Representative plaque assay showing reduced plaque sizes of the *i*Δ*znt* mutant grown in the presence of ATc (+ATc). (B) Quantification of plaque areas from three independent plaque assays (*n* = 3) showing that the reduced plaque size is significant. (C) Representative growth assay measuring the red fluorescence of parasites expressing tdTomato, showing that *i*Δ*znt* mutants supplemented with ATc (red lines) have a growth defect that is exacerbated in the presence of higher extracellular ZnSO_4_ concentrations. Controls without ATc are shown in blue. The concentration of ZnSO_4_ added to the culture is indicated in each graph (*n* = 3). (D) Quantification of parasite numbers at 6 days postinfection showing that parental and mutant parasites grow at a similar rate in the absence of added ZnSO_4_ (0 μM ZnSO_4_) but that there is a significant reduction in the growth of parasites lacking TgZnT when ZnSO_4_ is added to the medium (*n* = 3), *, *P* > 0.05; **, *P* > 0.01; ***, *P* > 0.01; n.s., not significant.

We investigated the role of TgZnT in parasite growth, and we performed plaque assays in the presence ATc and in the absence of ATc (Fig. 4A). Plaques were significantly smaller when parasites were grown in the presence of ATc (+ATc mutants) (Fig. 4A and B). We next wanted to investigate if the mutants were less able to cope with high extracellular concentrations of Zn^2+^, and for this we first transfected mutant parasites with a red fluorescent protein and selected the cells by fluorescence-activated cell sorting. These cells allowed us to study growth by following the red fluorescence as a function of time (Fig. 4C and D). We grew parasites (with and without ATc) in the presence of several concentrations of extracellular Zn^2+^ (Fig. 4C and D) up to 100 μM Zn^2+^, which did not show apparent toxicity to the host cells. Higher concentrations of Zn^2+^ were deleterious to the host cells (not shown). The growth results showed that the parental cell line grew fine at 1 to 10 μM Zn^2+^ and that only a small decrease was observed at 25 μM Zn^2+^. Higher concentrations of extracellular Zn^2+^ (75 to 100 μM) were deleterious to the growth of the parental cells. The +ATc mutants were intolerant to higher concentrations of Zn^2+^ and showed a clear and significant growth difference at 1, 10, and 25 μM Zn^2+^. At 75 μM Zn^2+^, the +ATc mutants were significantly deficient in their tolerance to Zn^2+^. Interestingly, the zinc-dependent growth difference between the +ATc mutants and mutants grown in the absence of ATc (−ATc mutants) was ablated in media devoid of Zn^2+^ supplementation (containing only contaminating Zn^2+^). These results support our hypothesis that TgZnT plays a role in the extracellular Zn^2+^ tolerance of T. gondii.

### TgZnT restores Zn^2+^ tolerance to Zn^2+^-sensitive yeast mutants.

**FIG 5:**
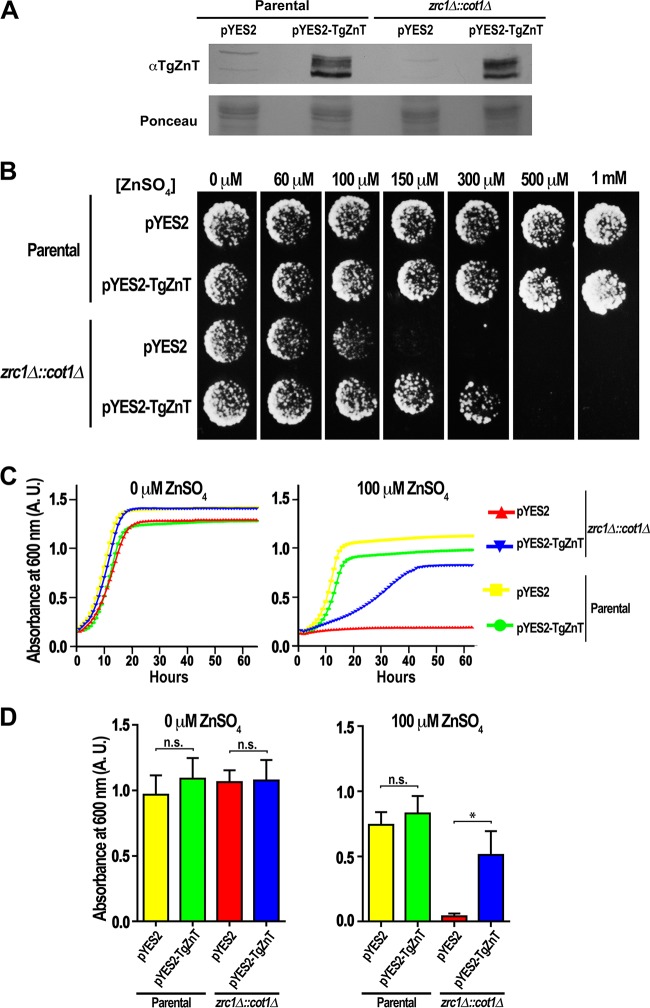
Expression of TgZnT restores Zn^2+^ tolerance to yeast. (A) Western blot analysis of lysates from parental and *zrc1*Δ::*cot1*Δ yeasts transformed with pYES2-TgZnT or pYES2 (empty vector) and grown in medium containing galactose for induction. Lysate from parental and *zrc1*Δ::*cot1*Δ yeasts transfected with pYES2-TgZnT shows labeling with anti-TgZnT. A portion of the Ponceau-stained membrane is shown as a loading control. (B) Representative example of agar plate growth assays (*n* = 3) showing that TgZnT expressing *zrc1*Δ::*cot1*Δ::*pYES2-TgZnT* mutants are capable of growth in medium supplemented with up to 300 μM ZnSO_4_, in contrast to the *zrc1*Δ::*cot1*Δ::*pYES2* mutant, which can grow only in medium supplemented with up to 100 μM ZnSO_4._ (C) Representative growth curves from 3 liquid culture assays show that *zrc1*Δ::*cot1*Δ mutants (blue) expressing TgZnT have increased tolerance to growth in medium supplemented with 100 μM ZnSO_4_, in contrast to *zrc1*Δ::*cot1*Δ mutants not expressing TgZnT (red). The parental cell lines transformed with pYES2 and pYES2-TgZnT are shown as controls (yellow and green curves, respectively). (D) Quantification of growth assay endpoints (65 h) showing a significant difference in the increased growth of *zrc1*Δ::*cot1*Δ mutants when complemented with TgZnT (*n* = 3). *, *P* > 0.05; n.s., not significant; A.U., absorbance units.

To investigate the Zn^2+^ transport function of TgZnT, we transformed *zrc1*Δ::*cot1*Δ yeast mutants, which lack their vacuolar zinc transporters and are unable to grow in media containing high concentrations of Zn^2+^, with a pYES2 expression plasmid containing the cDNA for *TgZnT* under the control of the galactose promoter. Western blot analysis of lysates from these mutants grown in media containing galactose showed labeling with anti-TgZnT (Fig. 5A), with the mutants showing a similar multiple-band profile with bands with sizes comparable to the ones observed in *Toxoplasma* lysates (Fig. 3D). Plate growth assays in the presence of different concentrations of ZnSO_4_ revealed that *zrc1*Δ::*cot1*Δ mutants expressing TgZnT were capable of tolerating higher concentrations of Zn^2+^ (up to 300 μM), whereas the *zrc1*Δ::*cot1*Δ mutants transfected with an empty vector tolerated only 100 μM (Fig. 5B). Assays in liquid media showed that the *zrc1*Δ::*cot1*Δ mutants transfected with the empty vector pYES2 were unable to grow (Fig. 5C and D, red line) in the presence of 100 μM Zn^2+^, while the expression of *TgZnT* in the *zrc1*Δ::*cot1*Δ mutants led to a partial growth recovery (Fig. 5C and D, blue line).

## DISCUSSION

We report that the gene *TgZnT*, present in the T. gondii genome, encodes the only annotated, functional Zn^2+^ transporter of the ZnT family in T. gondii and that this transporter is closely related to the ZnT2-like Zn^2+^ transporters found in plants. In extracellular tachyzoites, TgZnT localizes to large and small vesicles that, in electron microscopy images, were shown to fuse with large vacuoles, most likely the PLV. In intracellular tachyzoites, TgZnT localizes to vesicles that did not colocalize with either VP1 or T. gondii CRT. We hypothesize that these vesicles may be acidocalcisomes, which are similar to the zincosomes described in other cell types (19, 31). In this regard, there is evidence of the presence of large amounts of Zn^2+^ in acidocalcisomes of different species, as determined by X-ray microanalysis (32), and of Zn^2+^ transporters (ZnT) in acidocalcisomes of Trypanosoma cruzi (33) and T. brucei (34). Our laboratory previously determined the presence of Zn^2+^ in acidocalcisomes of *Toxoplasma* by X-ray microanalysis of whole cells (31, 35). It is likely that acidocalcisomes play a role in Zn^2+^ transport and trafficking in *Toxoplasma*. Preliminary data by our group suggest that a zinc transporter of another family (the ZIP family) that typically transports zinc into the cytoplasm (in the opposite direction of ZnT family transporters) localizes to these vesicles as well, lending credence to this hypothesis.

The mechanism responsible for the delivery of Zn^2+^ to the PLV or other compartments, where it would be required for the activity of metalloenzymes and other metalloproteins, has not been characterized in T. gondii or any other organism. It is possible that TgZnT distributes Zn^2+^ to various compartments as a way of activating apo-metalloenzymes, which are inactive in low-Zn^2+^ compartments. The distribution of Zn^2+^ to these compartments could be a mechanism for regulating the activity of these enzymes. There are numerous metalloproteases in T. gondii that are predicted to require Zn^2+^ as a cofactor for their activity (36–39), and due to the promiscuous activity of metalloproteases, they would require tight control in order to prevent unintended proteolytic activity. The presence or absence of the required Zn^2+^ cofactor would provide for potential regulation of their activity. The fusion of TgZnT vesicles or acidocalcisomes to the PLV in extracellular tachyzoites would also fit this model, as the PLV shares characteristics of a lysosome and proteolytic activity could be activated by the fusion of vesicles carrying Zn^2+^ and TgZnT at their membrane. The phosphorylation sites annotated in TgZnT may play a role in the regulation of transport activity, as was described for the human ZIP7 transporter (40).

In extracellular tachyzoites, our results support the hypothesis that TgZnT plays a role in the tolerance of T. gondii to the shift to high Zn^2+^ concentration upon egress. Our finding that the growth phenotype of the Δ*TgZnT* mutants was eliminated upon removal of supplementary Zn^2+^ from the media is the most significant support for this proposed role. The ability of TgZnT to restore Zn^2+^ tolerance when heterologously expressed in yeast mutants also provides support for its Zn^2+^-transporting function. Prior to egress, the dispersed nature of TgZnT vesicles in intracellular tachyzoites may allow for the rapid sequestration of Zn^2+^ throughout the tachyzoite upon egress and a subsequent trafficking of the vesicles to the PLV for final sequestration.

In summary, this report describes a functional Zn^2+^ transporter in T. gondii capable of rescuing Zn^2+^ tolerance upon heterologous expression in yeast mutants. TgZnT localizes to vesicles that fuse with the PLV, and its absence in T. gondii tachyzoites causes a Zn^2+^ concentration-dependent growth defect that becomes more pronounced with high concentrations of extracellular Zn^2+^. TgZnT is the first Zn^2+^ transporter to be characterized in an apicomplexan parasite, and its existence as the sole member of this family of Zn^2+^ transporters in these organisms suggests that its role may be conserved throughout the phylum.

## MATERIALS AND METHODS

### Gene identification and phylogenetic analysis.

A gene (TgGT1_251630, UniProt accession number S7V0D3) annotated as a member of the solute carrier 30 family and an ortholog of ZnT-2 (UniProt accession number Q9BRI3) was cloned and sequenced. The ORF of the annotated gene in the current version of ToxoDB encodes a protein of 896 amino acids with a predicted molecular weight of 97 kDa; however, we determined through sequencing and experimental evidence that the translation initiation site annotated in a previous version of ToxoDB (TGME49_chrXII:5,501,102) was the correct one.

### Generation of mutants.

For C-terminal tagging of the TgZnT gene, the 3′ 1,662 bp (minus the stop codon) of the gene annotated as a member of the solute carrier 30a2 family (slc30a2), TgGT1_251630, was amplified using primers P1 and P2 (see Table S2 in the supplemental material), which added the sequence required for ligation-independent cloning. The PCR product was purified using a Qiaex II gel extraction kit (Qiagen) and cloned into the pLIC-3×HA-CAT plasmid. The purified PCR product and plasmid were treated and combined as described by Huynh and Carruthers (25). Fifty micrograms of the sterilized plasmid pTgZnT-3×HA-CAT was transfected into 1 × 10^7^ RH Δ*ku80 TATi* parasites (41). Transfected parasites were selected with 20 μM chloramphenicol, and clones were isolated by limiting dilution. The genomic DNA of the clones was isolated and screened by PCR using a primer upstream of the original amplification from *TgZnT* (forward primer P3) and downstream pLIC-3×HA-CAT reverse primer P4 (Table S2). Clones were further confirmed by Western blot analysis.

10.1128/mSphere.00086-19.4 TABLE S2 Primers used for TgZnT work. Download Table S2, PDF file, 0.05 MB. Copyright © 2019 Chasen et al. 2019 Chasen et al. This content is distributed under the terms of the Creative Commons Attribution 4.0 International license.

Disruption of the *TgZnT* gene in RH was achieved by transfecting tachyzoites with 1 μg of pSAG1::CAS9-U6::sgUPRT (catalog number 54467; Addgene) (42), with the protospacer region being replaced with a protospacer (AGGAAGGCGTTTCCCCGTCC) near the 5′ end of the *TgZnT* coding region (modified with a New England Biolabs QuikChange site-directed mutagenesis kit by using primers P5 and P6) along with a separate dihydrofolate reductase (DHFR) drug selection cassette product generated via PCR. The parasites were selected with pyrimethamine followed by subcloning. Complementation/overexpression of *TgZnT* was accomplished by cloning the *TgZnT* gene, including the untranslated regions (UTRs) and potential promoter region, into the pCTH3 plasmid. The construct was transfected into Δ*znt* tachyzoites and selected using chloramphenicol, followed by subcloning.

For conditional knockdown of TgZnT, primers P13 and P14 were used to introduce the protospacer CGCGTCTTCAGCTCTCGCCT into pSAG1::CAS9-U6::sgUPRT, which then became pSAG1::CAS9-U6::sgZnT. Homology regions corresponding to the region upstream of the protospacer (TTGCTCTTTCGCTTCCTCTGCTCTGCGTTCGCTG) and the region at the beginning with the translational start codon (GCGGCTTGGCTGCGCCGCCGCGCTTCTTGGAACGCGGCAT) were added to a base primer (primers P15 and P16) to amplify the promoter insertion cassette (43). Four micrograms of linearized PCR product and 1 μg of pSAG1::CAS9-U6::sgZnT were transfected into RH Δ*ku80 TATi* parasites. Pyrimethamine (10 μM) was added to the transfection reaction mixture 24 h later, and the population was subcloned. Anhydrotetracycline (0.5 μg) was added to knock down the expression of ZnT.

### Parasite cultures and generation of mutants.

Tachyzoites of the T. gondii RH (25) and RH Δ*ku80 TATi* (43) strains were cultured in human telomerase reverse transcriptase (hTert) fibroblasts in Dulbecco’s modified Eagle’s medium (DMEM) supplemented with 1% fetal bovine serum, 1 mM sodium pyruvate, and 2 mM glutamine. Tachyzoites were obtained from infected hTert cells by passing them through a 25-gauge needle or otherwise collected from the supernatant of infected cells after natural egress. The RH Δ*ku80 TATi* strain was obtained from Boris Striepen (University of Georgia).

### Plaque and growth assays.

Plaque assays were performed as previously described (30) with modifications. For plaque growth assays, 125 tachyzoites were used for infection of hTert fibroblasts and allowed to grow for 10 days prior to fixing and staining. Growth assays of fluorescent cells were performed using TdTomato-expressing parasites in 96-well plates preseeded with hTert fibroblasts. Serum-free DMEM without phenol red was used for the growth assay, and ZnSO_4_ and ATc were added, when appropriate, along with 4,000 tachyzoites per well. The fluorescence (594 nm) from each well was recorded every 24 h for 8 days using a SpectraMax E^2^ plate spectrometer. A standard curve to determine parasite numbers was generated on the day of inoculation using known numbers of TdTomato-expressing parasites.

### TgZnT loop fusion expression and antibody production.

TgZnT-LF was constructed by cloning two loops (Fig. 1A) of the TgZnT cDNA using overlapping regions. The primers used were P9 and P10 for the first part of the fusion construct and P11 and P12 for the second part. The fusion protein was cloned into the PQE80L expression vector and transformed into E. coli. After induction with 1 mM IPTG (isopropyl-β-d-thiogalactopyranoside), soluble recombinant TgZnTLF was purified using a 1-ml HisPur nickel-affinity column (Thermo Fisher).

Antibodies against the recombinant TgZnT loop fusion protein (rTgZnT-LF) were generated in mice. Six CD-1 mice (Charles River) were inoculated intraperitoneally with 100 μg of rTgZnT-LF mixed with complete Freund adjuvant, followed by two boosts with 50 μg of rTgZnT-LF, with each boost being mixed with incomplete Freund adjuvant. The final serum was collected by cardiac puncture after CO_2_ euthanasia. The animal protocol used was approved by the UGA Institutional Animal Care and Use Committee (IACUC).

### Western blot analysis and immunofluorescence assays.

Purified tachyzoites were treated with cell lysis buffer M (Sigma) and 25 units of Benzonase (Novagen) for 5 min at room temperature, followed by addition of an equal volume of 2% SDS–1 mM EDTA solution. Total protein was quantified with a NanoDrop spectrophotometer (Thermo Scientific). Samples were resolved using a 10% bisacrylamide gel in a Tris-HCl–SDS buffer system (Bio-Rad). Gels were transferred for Western blot analysis. Primary antibody dilutions were as follows: 1:100 for anti-HA (monoclonal rat; Roche) and 1:1,000 for mouse anti-TgZnT. Secondary horseradish peroxidase-labeled antibodies were used at 1:10,000 dilutions.

Indirect immunofluorescence assays (IFA) were performed on either naturally egressed tachyzoites or infected hTert monolayers. Parasites or monolayers were washed once using buffer A with glucose (BAG; 116 mM NaCl, 5.4 mM KCl, 0.8 mM MgSO_4_, 50 mM HEPES, pH 7.2, 5.5 mM glucose) and then fixed with 3% formaldehyde for 15 min, followed by permeabilization using 0.25% Triton X-100 for 10 min and blocking with 3% bovine serum albumin. Labeling was performed as previously described (5). Images were collected using an Olympus IX-71 inverted fluorescence microscope with a Photometric CoolSnapHQ charge-coupled-device camera driven by DeltaVision software (Applied Precision, Seattle, WA). Superresolution images were collected using an Elyra S1 superresolution structured illumination microscopy system (Zeiss). The dilutions used were 1:2,000 for rabbit anti-VP1, 1:1,000 for anti-TgZnT, and 1:50 for rat anti-HA (Roche).

### Immunoelectron microscopy.

Extracellular T. gondii parasites endogenously expressing the C-terminal 3×HA tag (TgZnT-HA) were washed twice with phosphate-buffered saline (PBS) before fixation in 4% paraformaldehyde (Electron Microscopy Sciences, PA) in 0.25 M HEPES (pH 7.4) for 1 h at room temperature and then in 8% paraformaldehyde in the same buffer overnight at 4°C. Parasites were pelleted in 10% fish skin gelatin, and the gelatin-embedded pellets were infiltrated overnight with 2.3 M sucrose at 4°C and frozen in liquid nitrogen. Ultrathin cryosections were incubated in PBS and 1% fish skin gelatin containing mouse anti-HA antibody at a 1/5 dilution and then exposed to the secondary antibody, which was revealed with 10-nm protein–anti-gold conjugates. Sections were observed, and images were recorded with a Philips CM120 electron microscope (Eindhoven, the Netherlands) under 80 kV.

### Yeast zinc tolerance assays.

Parental and zinc-intolerant *zrc1*Δ::*cot1*Δ mutants (44) of Saccharomyces cerevisiae were transformed with the pYES2 empty vector or pYES2-TgZnT. Western blot analyses using mouse anti-TgZnT (1:1,000) were used to confirm expression in the pYES2-TgZnT-transformed cells. Yeast plate growth assays were performed on 1.5% agar plates containing a pH 6.5 complete supplement mixture lacking uracil (CSM−Ura; Sunrise Science) supplemented with 2% galactose and adjusted to various concentrations of Zn^2+^ using ZnSO_4_. Assays were performed using 3 × 10^5^ yeast cells per 10-μl droplet and imaged after 48 h of growth.

Liquid growth assays were performed as described by Stasic et al. (8) with modifications. Yeast cells were grown on 96-well plates in CSM−Ura with 2% galactose that was either supplemented with 100 μM ZnSO_4_ or not supplemented. Each well was inoculated with 6 × 10^6^ yeast cells in 200 μl. Readings were performed every hour using a BioTek Synergy H1 hybrid tester.

### Statistical analyses, modeling, alignments, and tree generation.

All statistical analyses were performed using GraphPad Prism software (version 7). Modeling was performed using the Phyre2 server (45). Alignments were performed using the T-Coffee multiple-sequence alignment server (46) and manually trimmed to remove gaps. Trees were generated using the software Geneious and the Juke-Cantor algorithm and bootstrapped (100 cycles) to generate the consensus tree.
