# Supplementary figures and images for: The Vacuolar Zinc Transporter TgZnT Protects Toxoplasma gondii from Zinc Toxicity
Source: mSphere. 2019 May 22;4(3):e00086-19. doi: 10.1128/mSphere.00086-19 (PMC6531880; doi:10.1128/mSphere.00086-19)

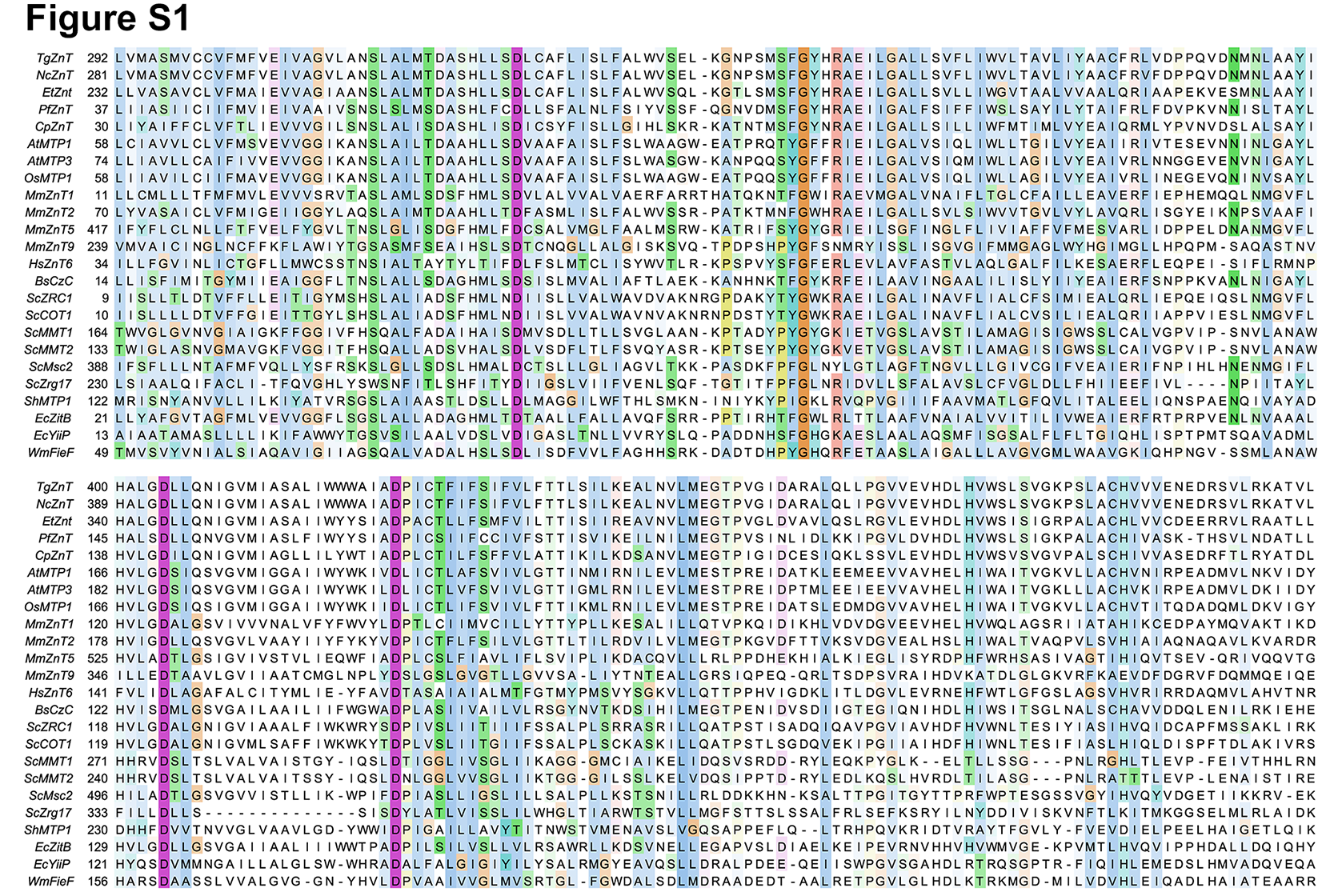

Supplement: FIG S1 [file mSphere.00086-19-sf001.tif]

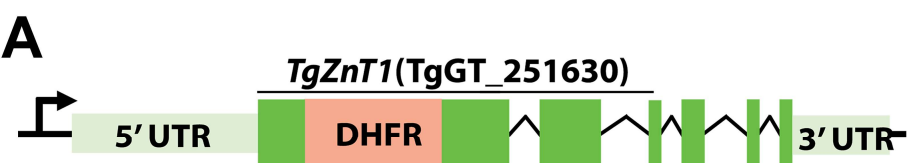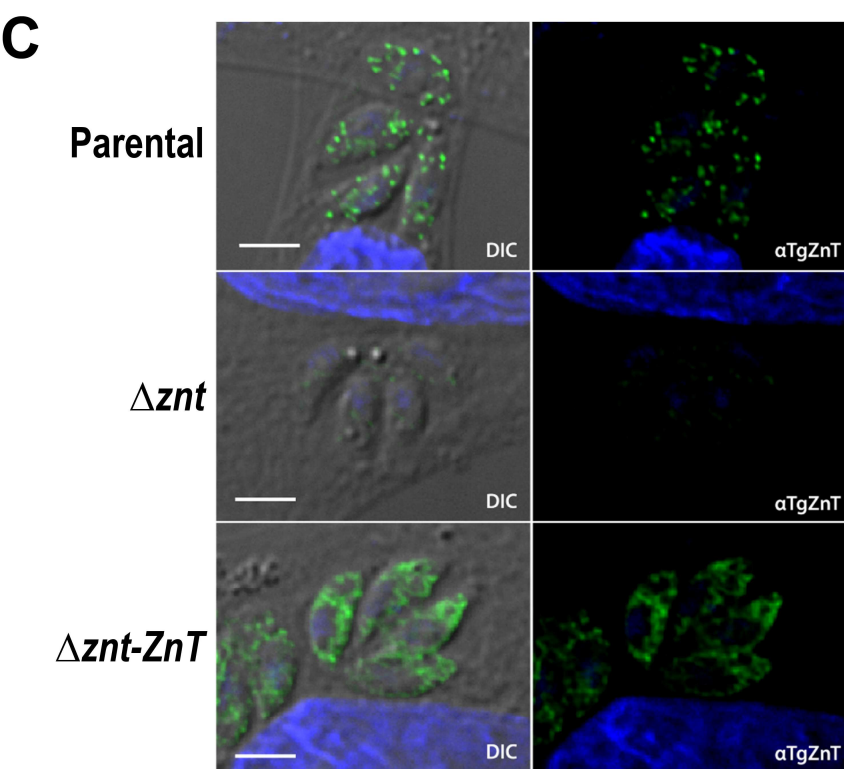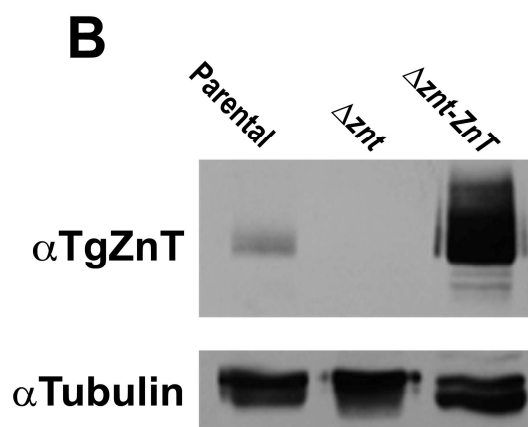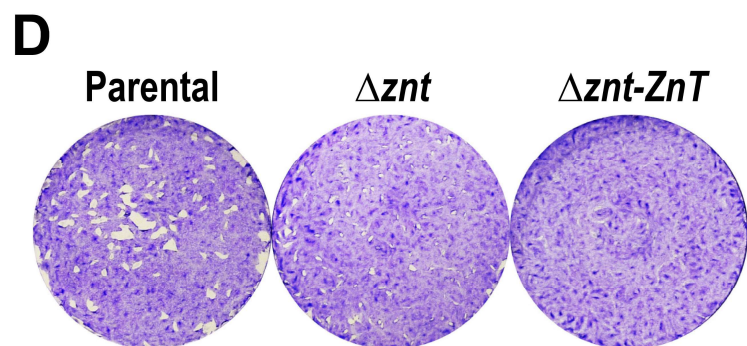

Supplement: FIG S2 [file mSphere.00086-19-sf002.pdf]
