# Supplementary material for: The Vacuolar Zinc Transporter TgZnT Protects Toxoplasma gondii from Zinc Toxicity
Source: mSphere. 2019 May 22;4(3):e00086-19. doi: 10.1128/mSphere.00086-19 (PMC6531880; doi:10.1128/mSphere.00086-19)
Supplement: TABLE S1 [file mSphere.00086-19-st001.pdf]

Table S1 Amino Acid Sequences Used for Alignments and Tree Generation

Organism, Protein Name	UniProtID	EuPathDB ID
Toxoplasma gondii GT1, TgZnT	S7V0D3	TGGT1_251630
Neospora caninum, NcZnT	F0VR98	NCLIV_066710
Eimeria tenella, EtZnT	U6L3J7	ETH_00033120
Plasmodium falciparum, PfZnT	Q8IBU1	PF3D7_0715900
Cryptosporidium parvum, CpZnT	Q5CSE6	cgd1_3050
Arabidopsis thaliana, AtMTP1	Q9ZT63	
Arabidopsis thaliana, AtMTP3	Q9LXS1	
Oryza sativa, OsMTP1	Q688R1	
Mus musculus, MmZnT1	Q60738	
Mus musculus, MmZnT2	Q2HJ10	
Mus musculus, MmZnT5	Q8R4H9	
Mus musculus, MmZnT9	Q5IRJ6	
Homo sapiens, HsZnT6	Q6NXT4	
Bacilus subtilis, BsCzC	O07084	
Saccharomyces cerevisiae, ScZRC1	P20107	
Saccharomyces cerevisiae, ScCOT1	P32798	
Saccharomyces cerevisiae, ScMMT1	Q03218	
Saccharomyces cerevisiae, ScMMT2	Q08970	
Saccharomyces cerevisiae, ScMsc2	Q03455	
Saccharomyces cerevisiae, ScZrg17	P53735	
Stylosanthes hamata, ScMTP1	Q84ND6	
Eschericha coli, EcZitB	P75757	
Eschericha coli, EcYiiP	P69380	
Wautersia metallidurans, WmFieF	Q1LHU8	
