# Supplementary material for: The Vacuolar Zinc Transporter TgZnT Protects Toxoplasma gondii from Zinc Toxicity
Source: mSphere. 2019 May 22;4(3):e00086-19. doi: 10.1128/mSphere.00086-19 (PMC6531880; doi:10.1128/mSphere.00086-19)
Supplement: TABLE S2 [file mSphere.00086-19-st002.pdf]

Table S2: Primers Used for TgZnT Work

Nº	Primer
1	5´_tacttccaatccaatttaatGCCTTCTCTCTCGTCGATGTTCACTGGGGAATG_3´
2	5´_ tcctccacttccaattttagcGCGGCGGAAAACTTTCATGGG_3´
3	5´_ ACTGGGCTATCGCCGACCCAATTTG_3´
4	5´_ AAGTTAGGAAGGCGTTTCCCCGTCCG_3´
5	5´_ AAAACGGACGGGGAAACGCCTTCCTA_3´
6	5´_ctcgagTATGCCAACAGTATTCAAGAAAACGAGGGC_3´
7	5´_ TTCGCTTCCTCTGCTCTGCGTTCGCTGCCTcagcacgaaaccttgcattcaaac_3´
8	5´_ CCGCCGCGCTTCTTGGAACGCGGCATGGCGggttgaagacagacgaaagcagttg_3´
9	5´_ ggatcctCCGCCCTCAGCGTTCT_3´
10	5´_ GTCGACCTCGGGAAAGGA_3´
11	5´_ tcctttcccgaggtcgacGTCGACGGCGAACTGATGTTC_3´
12	5´_ ctcgagCTACTCGAGACGGACGTAGATGTC_3´
13	5´_ tgaagacgcgGTTTTAGAGCTAGAAATAGC_3´
14	5´_ gctctcgcctAACTTGACATCCCCATTTAC_3´
15	5´_TTGCTCTTTCGCTTCCTCTGCTCTGCGTTCGCTGaactacgtggacattaagttccattc_3´
16	5´_GCGGCTTGGCTGCGCCGCCGCGCTTCTTGGAACGCGGCATggttgaagacagacgaaagcag _3´
